# Radiographic Changes Following Regenerative Endodontic Treatment in Immature Permanent Teeth with Traumatic or Carious Pulp Necrosis: A Retrospective Study

**DOI:** 10.3390/jfb17090445

**Published:** 2026-09-03

**Authors:** Carolina Caleza-Jiménez, María Biedma-Perea, Marcela Arenas-González, Adrián Curto-Aguilera, Asunción Mendoza-Mendoza, David Ribas-Pérez

**Affiliations:** 1 Pediatric Dentistry Division, Department of Stomatology, School of Dentistry, University of Seville, C/Avicena s/n, 41009 Seville, Spain; ccaleza@us.es (C.C.-J.); amendoza@us.es (A.M.-M.); dribas@us.es (D.R.-P.); 2 Department of Surgery, Faculty of Medicine, University of Salamanca, Avenue Universidad de Coimbra s/n, 37007 Salamanca, Spain; adrian_odonto@usal.es

**Keywords:** regenerative endodontic, immature teeth, necrosis

## Abstract

**Objective:** To compare radiographic changes following regenerative endodontic procedures in immature permanent teeth with pulp necrosis of traumatic or carious origin. **Methods:** A retrospective study was conducted in 20 immature permanent teeth from 20 patients (10 traumatic and 10 carious cases) treated with a standardized regenerative endodontic protocol. Root length, dentinal wall thickness, periapical status, and type of repair were assessed using standardized periapical radiographs at baseline and 12 months. Radiographs were independently evaluated by two investigators blinded to etiology, and inter-examiner agreement for type of repair was assessed using Cohen’s kappa. **Results:** No statistically significant differences were found in root-length change between the groups. A statistically significant greater increase in dentinal wall thickness was observed in the carious group compared with the traumatic group; however, the groups differed substantially in tooth type. No statistically significant differences were observed in Periapical Index scores after 12 months of follow-up, and periapical healing (PAI 1–2) was observed in all cases. Periodontal-associated repair was observed in all traumatic cases, whereas pulpal-associated repair was observed in all carious cases. **Conclusions:** Regenerative endodontic procedures were associated with favorable radiographic outcomes in immature permanent teeth with pulp necrosis of traumatic or carious origin. However, the small sample size, short follow-up, absence of a control group, and substantial differences in tooth type between etiological groups limit causal interpretation of the observed differences.

## 1. Introduction

Traditionally, immature permanent teeth with pulp necrosis have been treated using apexification techniques, whose objective was to achieve artificial apical closure without allowing continued root development. This procedure was performed for many years using calcium hydroxide, a material that, due to its progressive resorption and loss of effectiveness, required multiple replacements over time, especially in teeth with limited root development. Subsequently, the introduction of biomaterials such as mineral trioxide aggregate (MTA) made it possible to reduce the time required for the formation of this apical barrier [1].

Although these techniques achieved resolution of the infectious process and elimination of clinical signs and symptoms, the tooth remained with thin dentinal walls and was structurally fragile, significantly increasing the risk of root fracture in the long term [2]. In recent years, regenerative endodontic procedures (REPs) have become an established therapeutic alternative for immature permanent teeth with pulp necrosis, as they may allow continued root development and improve the structural characteristics of the tooth. These procedures are based on adequate disinfection of the root canal system and the induction of a biological scaffold, promoting tissue repair and continued root development [3,4].

In this context, both the American Association of Endodontists (AAE) [5] and the European Society of Endodontology (ESE) [6] have proposed standardized clinical protocols for performing these procedures. In the present study, the protocol recommended by ESE [5] was used as the primary reference because of its conservative approach, emphasis on biological principles, and clinical applicability in immature permanent teeth with pulp necrosis.

The success of regenerative endodontic procedures in immature permanent teeth with pulp necrosis depends, at least in part, on the preservation of the biological potential of the apical complex, including the apical papilla and Hertwig’s epithelial root sheath, which are key structures for continued root development [7]. Experimental studies have demonstrated that Hertwig’s epithelial root sheath plays a fundamental role in odontoblastic differentiation and root morphogenesis, and that its damage may lead to interruption of root growth and the formation of short roots with an open apex [8,9].

Furthermore, dental trauma, particularly injuries involving tooth displacement, may cause damage to the periodontal and periapical tissues and potentially compromise subsequent root development [1,7]. From a biological perspective, these observations raise the possibility that teeth with pulp necrosis of traumatic origin may exhibit different patterns of repair and root development compared with teeth in which necrosis results from a carious process. Previous studies have suggested that differences in the extent and nature of tissue injury may influence the biological response following regenerative endodontic procedures [10].

However, despite these differences in the etiopathogenic mechanisms, the available clinical literature shows variable and, in some cases, contradictory results regarding the influence of the etiology of pulp necrosis on the outcomes of regenerative endodontic procedures [11]. Therefore, the aim of the present study was to compare radiographic changes following regenerative endodontic procedures in immature permanent teeth with pulp necrosis of traumatic or carious origin, focusing on changes in root length, dentinal wall thickness, periapical status, and type of repair. The hypothesis was that the etiology of pulp necrosis would be associated with differences in these radiographic outcomes.

## 2. Materials and Methods

### 2.1. Data Collection

Data were collected from patients treated at a private dental clinic in Seville between January 2021 and December 2025. The patients were between 7 and 8.5 years of age and presented with immature permanent teeth with pulp necrosis that underwent REPs. Cases that met the inclusion criteria were assigned a study subject number, and data were extracted from their clinical records (Table 1).

This retrospective observational study involved the secondary analysis of pre-existing clinical and radiographic records. No additional interventions, examinations, procedures, or patient contact were performed for research purposes. Before analysis, eligible cases were de-identified and assigned a study-specific identification code, and no names or other directly identifying information were included in or accessible through the research dataset. Access to the original clinical records was restricted to authorized clinical personnel. The study was conducted in accordance with applicable ethical and data-protection principles. According to the guidance of the Ethics Committee of the University of Seville (CEIUS), formal ethical review was not required for this retrospective study because it involved the analysis of pre-existing clinical and radiographic records without additional interventions or patient contact. Informed consent for routine clinical care had been obtained from the parents or legal guardians of the pediatric patients and included authorization for the anonymized use of clinical and radiographic data for research purposes. The study was conducted in accordance with the ethical principles of the Declaration of Helsinki.

**Figure 1:**
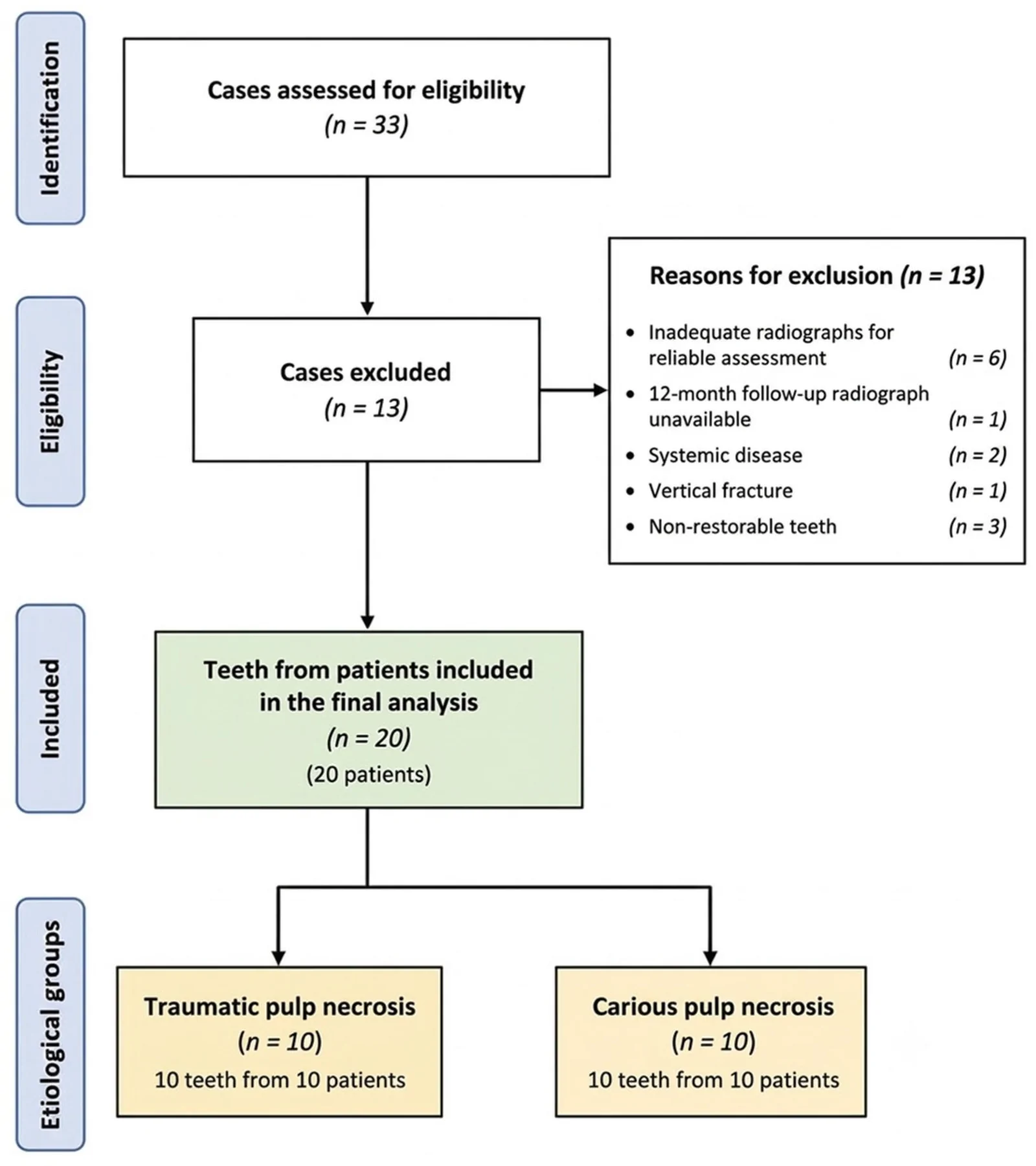
Flow diagram of patient and tooth selection for the retrospective study.

Of the 33 cases initially assessed for eligibility, 13 were excluded according to the predefined inclusion and exclusion criteria. Six cases were excluded because the available radiographs were inadequate for reliable radiographic assessment, one because the 12-month follow-up radiograph was unavailable, two because the patients had systemic diseases, one because of a vertical fracture, and three because the teeth were non-restorable. The remaining 20 teeth from 20 patients met the eligibility criteria and were included in the final analysis. These cases were classified according to the etiology of pulp necrosis into two groups: traumatic etiology (*n* = 10) and carious etiology (*n* = 10). All 20 included cases had adequate radiographic follow-up at 12 months. A retrospective analysis of radiographic outcomes was subsequently performed (Figure 1).

### 2.2. Treatment Protocol

All teeth were treated by the same operator and isolated using a rubber dam. Local anesthesia without vasoconstrictor was administered at the first visit, followed by access cavity preparation and working length determination. No mechanical instrumentation of the root canal walls was performed. The root canals were irrigated with approximately 20 mL of 1.5–2.5% sodium hypochlorite over approximately 5–10 min. The irrigation needle was positioned 1–2 mm short of the working length to minimize the risk of periapical extrusion. A final irrigation with 17% EDTA was subsequently performed.

An intracanal medicament was then placed using the triple antibiotic paste described by Banch and Trope [12], consisting of 250 mg of ciprofloxacin, 400 mg of metronidazole, and 50 mg of minocycline mixed in a 1:1:1 ratio. The antibiotics were mixed with propylene glycol to obtain an intracanal paste. The medicament was maintained in the root canal for 4 weeks, and the access cavity was temporarily restored.

At the second visit, the intracanal medicament was removed, and bleeding was induced into the root canal using a size 25 hand file inserted 1–2 mm beyond the apical foramen to promote the formation of a blood clot. ProRoot MTA (Dentsply Sirona, York, PA, USA) was then placed to a thickness of approximately 2–3 mm. A conventional high-density glass-ionomer cement was placed over the MTA as an intermediate base. The definitive coronal restoration was performed during the same visit using a two-step total-etch adhesive system (OptiBond Solo Plus, Kerr, Brea, CA, USA) and a microhybrid composite resin (Filtek Z250, 3M, Saint Paul, MN, USA), applied using a layering technique.

### 2.3. Radiographic Evaluation

Preoperative and follow-up periapical radiographs were obtained using a standardized paralleling technique with the Rinn XCP alignment system (Dentsply Sirona Inc., York, PA, USA). Follow-up radiographs were obtained with the aim of reproducing the same projection geometry and angulation as the preoperative images. The images were stored in JPEG format and transferred to Microsoft PowerPoint for radiographic analysis. A static ruler with a known reference scale was used to calibrate the images, and the same reference scale and measurement procedure were consistently applied to all radiographs. The same anatomical reference points, measurement definitions, and PowerPoint guides were used consistently across preoperative and follow-up radiographs.

Root length, dentinal wall thickness, and the size of periapical periodontitis were then measured using PowerPoint guides. Because radiographs were obtained at different clinical time points, minor differences in projection geometry and magnification could not be completely excluded.

The type of repair was classified according to the anatomical location and pattern of newly formed hard tissue and its relationship with the root canal space, dentinal walls, external root surface, and periodontal tissues. Pulpal-associated repair was defined as radiographic evidence of hard-tissue deposition and continued root development occurring within the root canal space, including progressive thickening of the dentinal walls and/or narrowing of the root canal. Periodontal-associated repair was defined as hard-tissue formation occurring predominantly along the external root surface or at the level of the periodontal tissues, without evidence of continuous internal dentinal deposition within the root canal.

The images were evaluated independently by two investigators (CC and MB), who were blinded to the etiology of pulp necrosis. Inter-examiner agreement was assessed using Cohen’s kappa (κ = 0.70). In cases of disagreement, consensus was reached through discussion between the two investigators.

Apical lesions were quantified and classified using the Periapical Index (PAI) [13], which represents different stages of apical periodontitis. The PAI was scored from 1 to 5, where 1 = normal periapical structures; 2 = minor changes in bone structure; 3 = changes in bone structure with some mineral loss; 4 = periodontitis with a well-defined radiolucent area; and 5 = severe periodontitis with exacerbating features.

The categories proposed by Friedman and Mor [14] were modified to interpret treatment outcomes as follows: healed (PAI 1–2, without signs of resorption/calcification); healing (PAI 3–4, with improvement in follow-up radiographs and no signs of resorption/calcification); or diseased (increasing or unchanged PAI scores, as observed in follow-up radiographs after treatment, or with signs of resorption/calcification).

Quantification of changes in root dimensions was performed by determining variations in root length and width. Root length was measured as a straight line from the cementoenamel junction (CEJ) to the radiographic apex of the tooth. Root width was defined as the total buccolingual dimension of the root measured at the standardized measurement level, whereas root canal width was defined as the buccolingual dimension of the root canal space at the same level. The remaining dentinal wall thickness was calculated as the difference between root width and root canal width. This value represents the combined thickness of the buccal and lingual dentinal walls.

**Figure 2:**
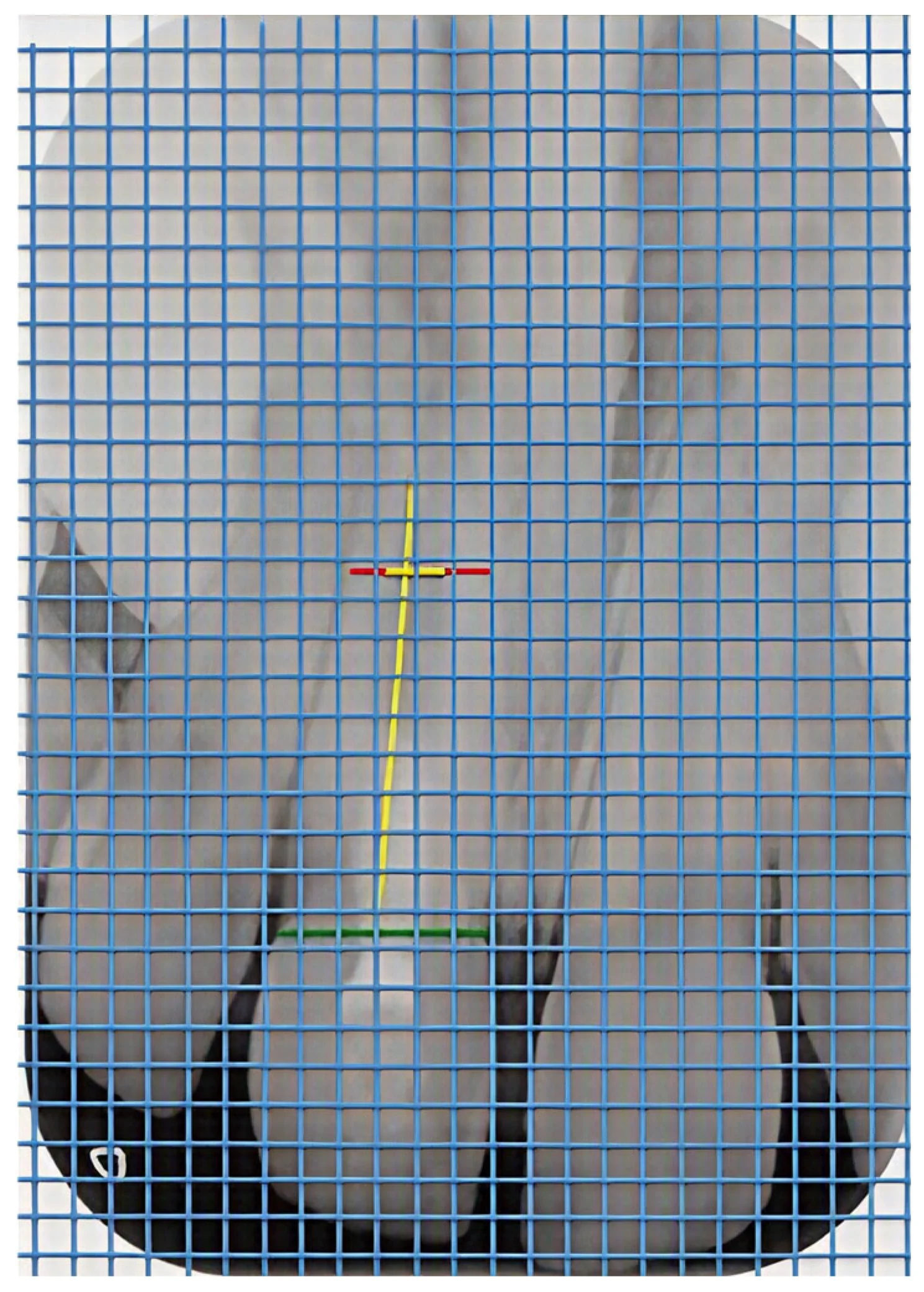
Measurement of the differences in root length and dentin wall thickness from the radiographs. The vertical yellow line denotes the distance from the cementoenamel junction (CEJ) to the radiographic apex of the tooth. The horizontal yellow line represents the distance between the internal dentinal walls at the apex, the red line measures the distance between the external dentinal walls at the apex, while the green line denotes the same measurement at the CEJ.

Differences in root length and dentinal wall thickness between preoperative and follow-up radiographs were calculated as percentage changes (Figure 2).

Radiographic assessment of the type of repair was based on the observed pattern of hard-tissue formation and root development, considering the biological mechanisms of dentin/cementum formation and periodontal tissue involvement described in previous studies [8,9].

### 2.4. Statistical Analysis

Statistical analyses were performed using SPSS version 25 (IBM Corp., Armonk, NY, USA). Given the small sample size, descriptive statistics were used to summarize the clinical and radiographic characteristics of the study groups. The Shapiro–Wilk test was used to assess the distribution of continuous variables.

The Mann–Whitney U test was used to compare percentage changes in root length and dentinal wall thickness, as well as PAI scores, between the traumatic and carious groups because the sample size was small and the data did not demonstrate a normal distribution. Fisher’s exact test was used to assess associations involving categorical variables because of the small sample size and limited expected cell counts. Spearman’s rank correlation coefficient was used to evaluate the association between changes in root length and dentinal wall thickness.

A two-sided *p*-value < 0.05 was considered statistically significant. No multivariable adjustment was performed because of the small sample size and the substantial imbalance in tooth type between the etiological groups. In particular, all traumatic cases involved maxillary central incisors, whereas the carious group consisted predominantly of molars, making reliable adjustment for tooth type infeasible.

## 3. Results

### 3.1. Sample Characteristics

During the 2021–2025 study period, a total of 20 immature non-vital teeth underwent REPs. The follow-up period was 12 months. Of the 20 teeth, 10 were of traumatic etiology and 10 of carious origin. Ten teeth were incisors, while the remaining 10 were molars. All incisors were maxillary central incisors (11 or 21). Among the molars, two were maxillary and the rest mandibular. The age range of the patients was 7–8 years. Regarding the preoperative stage of root development, Cvek stages 3 and 4 predominated in caries cases, while stages 2 and 3 were more frequent in trauma cases (Table 2 and Table 3).

Inter-examiner agreement for the radiographic assessment was assessed using Cohen’s kappa, yielding a κ value of 0.70, indicating substantial agreement between the two investigators.

### 3.2. Analysis of Changes in Root Dimensions

**Figure 3:**
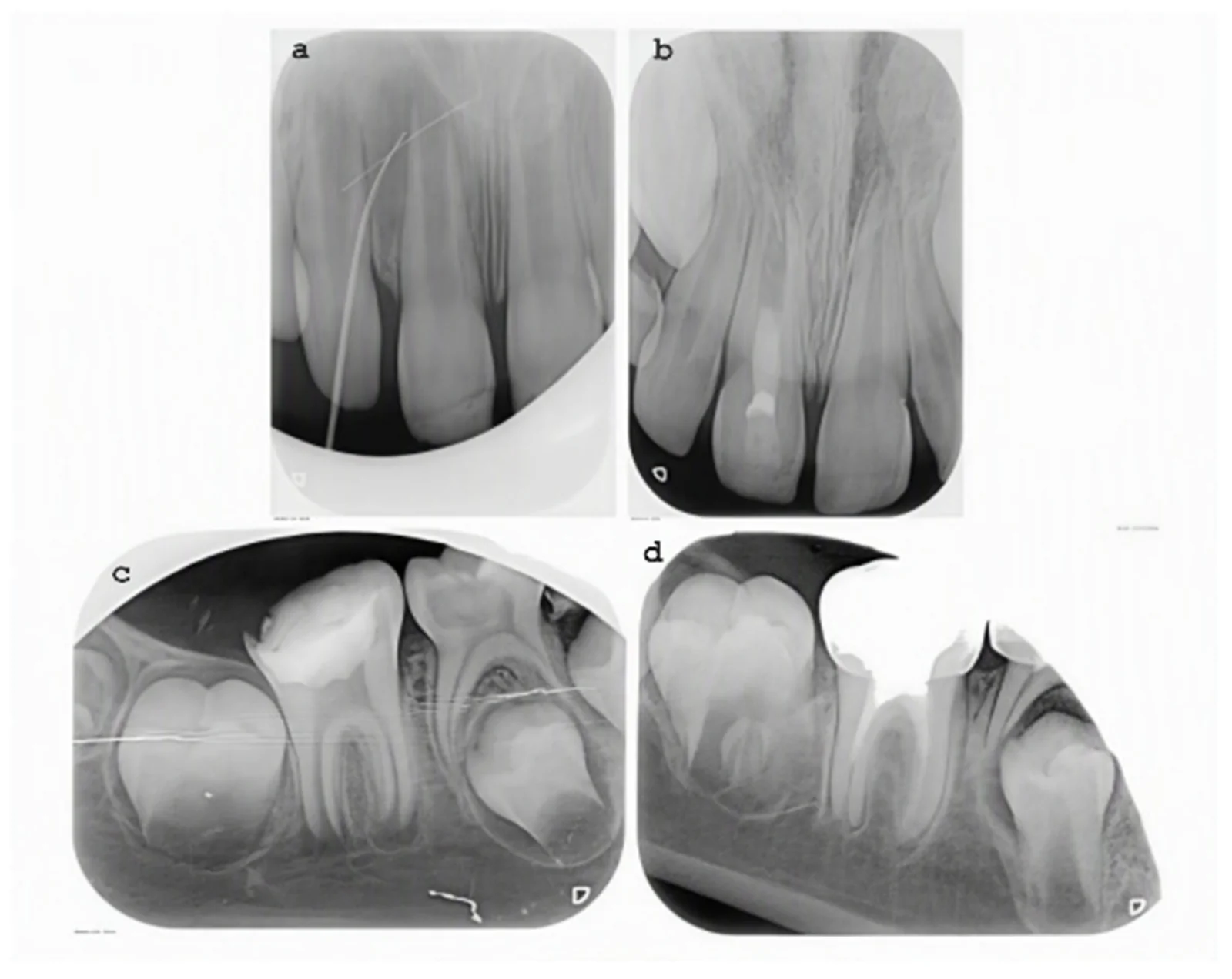
Radiographic apical healing following regenerative endodontic treatment. Necrotic maxillary incisor with traumatic etiology (**a**) and 12 months after treatment (**b**). Necrotic mandibular first molar with carious etiology (**c**) and 12 months after treatment (**d**).

At the 12-month postoperative radiographic evaluation, the overall sample showed a mean increase of 12.95% in root length (SD 10.60%) and 34.35% in width (SD 25.78%) (Figure 3).

Among incisors (trauma group), the mean increase in root length was 9.92% (SD 11%), and in width 19.76% (SD 11.76%). In molars (caries group), the mean increase in root length was 15.97% (SD 9.79%) and in width 48.94% (SD 28.13%) (Table 2 and Table 3). The relationship between changes in root length and dentinal thickness according to etiology was analyzed using the non-parametric Mann–Whitney U test, as the Shapiro–Wilk test indicated a non-normal distribution. No statistically significant differences were found in the percentage change in root length between the traumatic and carious etiology groups (U = 30, *p* = 0.130).

However, statistically significant differences were found in the percentage change in dentinal thickness (U = 21, *p* = 0.028). Rank analysis showed that the caries group had a higher mean rank (13.40 vs. 7.60), indicating that teeth treated for carious etiology (molars) exhibited greater increases in dentinal thickness compared to those of traumatic origin.

The relationship between the two continuous variables (root length and dentinal thickness) was assessed using Spearman’s correlation test. The rho value was −0.037, indicating no correlation between the variables. Additionally, the *p*-value was 0.876 (*p* > 0.05), confirming that the relationship was not statistically significant. Thus, changes in root length and dentinal thickness can be considered independent.

### 3.3. Analysis of Periapical Healing

No statistically significant differences were observed between the two groups in terms of PAI scores after 12 months of follow-up (*p* > 0.05). Both groups showed high success rates, with no significant differences between them, although different healing patterns were observed. Furthermore, no significant differences were found between root development stages in the analysis of final outcomes.

## 4. Discussion

REPs have become established as a treatment modality for immature permanent teeth with pulp necrosis in pediatric populations [15]. Their main objectives are the resolution of periapical pathology and the continuation of root development. This clinical study aimed to evaluate radiographic outcomes following REPs according to the etiology of pulp necrosis, primarily comparing traumatic versus carious origin.

Pulp necrosis is a pathological condition characterized by the death of dental pulp tissue due to various etiological factors, primarily those analyzed in this study: caries-related infection and dental trauma. Bacteria responsible for dental caries penetrate the dental tissues and reach the pulp, eliciting an inflammatory response that, if left untreated, may progress to necrosis. In addition, dental trauma—both acute and chronic—is an important etiological factor, particularly among young patients and athletes. Traumatic ischemia may disrupt the blood supply to the pulp and ultimately lead to necrosis. Other etiological factors reported in the literature include developmental anomalies, such as dens evaginatus [11,16], as well as dental hypoplasia [17].

Regarding radiographic changes, the present study found a significantly greater increase in dentinal wall thickness in the carious group than in the traumatic group. However, this finding should be interpreted cautiously because the etiological groups differed substantially in tooth type and stage of root development. Previous studies, such as that by Theekakul et al. [18], included different etiologies and reported better outcomes in younger patients with non-traumatic cases, suggesting that patient age may also be an important prognostic factor because of the potentially greater regenerative potential in younger patients. Similarly, Hu et al. [11] and Zeng et al. [16] observed greater root growth and increased dentinal thickness in teeth with developmental anomalies, such as dens evaginatus, compared with dental trauma cases. These findings provide a possible context for the differences observed in the present study; however, differences in patient characteristics, tooth type, developmental stage, and etiology between studies should be considered when making comparisons.

In contrast, the traumatic group showed greater variability in outcomes. The study by Cheng et al. [19], conducted on traumatized incisors, reported a success rate of 80.6%, identifying avulsion as a factor associated with failure. Meanwhile, Wikström et al. [20] reported a success rate of 60%, highlighting the difficulty in inducing intracanal bleeding as a limiting factor. These findings suggest that the severity and type of trauma, as well as the extent of damage to the periapical structures, may influence regenerative outcomes. In the present study, however, the traumatic cases included lateral luxation, subluxation, extrusive luxation, and coronal fractures, with no cases of avulsion. Therefore, the relatively favorable outcomes observed in the traumatic group may partly reflect the specific spectrum of traumatic injuries represented in this cohort.

The biological mechanisms underlying the differences observed between the etiological groups remain uncertain. Previous experimental and clinical studies have suggested that the preservation and biological activity of the apical complex, including the apical papilla and Hertwig’s epithelial root sheath, may contribute to continued root development following regenerative endodontic procedures [7,8,9]. In teeth affected by trauma, injury to the periodontal and periapical tissues could potentially influence these structures and thereby affect subsequent root maturation. However, the apical papilla and Hertwig’s epithelial root sheath were not directly assessed in the present study. Therefore, their involvement cannot be established from our data and should be regarded as a possible biological hypothesis rather than a demonstrated mechanism.

Other biological factors may also contribute to differences in regenerative outcomes. For instance, studies such as Jiang et al. [21] have shown that an inflammatory environment can negatively affect stem cell differentiation, thereby potentially impairing regenerative capacity. Similarly, Palma et al. [22] suggest that the survival and functionality of the apical papilla may be compromised in the presence of periapical pathology, potentially limiting regenerative outcomes in teeth with trauma-induced necrosis. Nevertheless, these mechanisms were not directly evaluated in the present study and therefore cannot be considered explanations established by our findings.

Overall, all 20 treated teeth were available for radiographic evaluation at the 12-month follow-up. Previous studies have reported favorable clinical outcomes following regenerative endodontic procedures in immature permanent teeth with pulp necrosis. For example, Pereira et al. [1] reported a clinical success rate of 95.5%, while Nazzal et al. [23] described favorable outcomes after a 2-year follow-up period. Kahler et al. [24] also reported a high rate of periapical resolution. These findings provide context for the favorable radiographic outcomes observed in the present study; however, direct comparisons should be interpreted cautiously because of differences in study design, sample characteristics, outcome definitions, and follow-up periods.

Our results showed that overall healing of apical periodontitis, defined as PAI scores representing healthy or nearly healthy periapical status (scores 1 and 2), occurred in 100% of cases. However, Wikström et al. [20] observed that, in cases where severe trauma was present, apical periodontitis persisted in 63%.

The 100% healing rate observed in the present cohort, including all traumatic cases, should be interpreted in the context of the characteristics of the study sample. The traumatic group consisted of teeth affected by lateral luxation, subluxation, extrusive luxation, and coronal fractures, with no cases of avulsion. Consequently, the traumatic injuries represented in this cohort may not reflect the full spectrum or severity of traumatic injuries affecting immature permanent teeth. The absence of avulsion and other potentially more severe traumatic injuries may have contributed to the favorable healing outcomes observed. In addition, the relatively small sample, predefined inclusion and exclusion criteria, and single-operator setting may have reduced clinical variability and contributed to the high healing rate. Therefore, these findings should not be generalized to all immature permanent teeth with traumatic pulp necrosis.

A key factor influencing outcomes is the clinical protocol used during regenerative endodontic procedures. In all cases in this study, irrigation with sodium hypochlorite was performed, followed by placement of a triple antibiotic paste (ciprofloxacin, metronidazole, and minocycline). At the second visit, a blood clot was induced within the root canal, followed by the placement of MTA. Major guidelines, such as those proposed by the AAE [5] and the ESE [6], outline different aspects of canal disinfection, intracanal medication, and biological scaffolding. Consequently, recent studies in the literature show heterogeneity in the application of these protocols. Methodological differences among studies may explain the variability in clinical and radiographic findings and make direct comparisons difficult.

The use of triple antibiotic paste (TAP), consisting of ciprofloxacin, metronidazole, and minocycline, was selected based on its established use in regenerative endodontic procedures and its broad antimicrobial spectrum. Adequate disinfection of the root canal system is an important prerequisite for regenerative procedures, particularly in immature teeth with pulp necrosis. However, the use of minocycline in TAP has been associated with the risk of dental discoloration, which represents a relevant limitation, particularly in anterior teeth. In the present study, no clinically evident crown discoloration was observed in any of the treated teeth during the 12-month follow-up period. Nevertheless, the possibility of discoloration cannot be completely excluded, particularly over longer follow-up periods. Future protocols may consider alternative antibiotic combinations or medicaments without minocycline when esthetic considerations are particularly relevant.

Despite this variability, there is general consensus that regenerative procedures may allow resolution of periapical pathology and, in many cases, promote continued root development.

Regarding the limitations of this study, the small sample size, with only 10 teeth per group, limits the statistical power to detect small or moderate between-group differences and increases the uncertainty of the effect estimates. Therefore, the absence of a statistically significant difference in root-length change should not be interpreted as evidence of equivalence or as demonstrating the absence of a clinically meaningful difference. In addition, the relatively short follow-up period of 12 months does not allow for long-term evaluation, as some published studies have reported follow-up periods of up to 30 or 36 months. Therefore, the favorable outcomes observed in the present study should be interpreted with caution.

An important additional limitation is the unequal distribution of tooth types between the etiological groups. All traumatic cases involved maxillary central incisors, whereas the carious group consisted predominantly of molars. Consequently, the observed differences in root development and dentinal wall thickening may be influenced by anatomical and developmental differences between tooth types rather than by the etiology of pulp necrosis alone. Because of the retrospective design and the lack of sufficient overlap in tooth types between the groups, comparisons within the same tooth type or statistical adjustment for tooth type were not feasible. Therefore, the differences observed between the traumatic and carious groups cannot be attributed exclusively to the etiology of pulp necrosis. The stage of root development also differed between the groups, with Cvek stages 2 and 3 predominating in the traumatic group and stages 3 and 4 in the carious group. In addition, the severity and type of trauma may have influenced the observed outcomes. These factors could not be adequately separated from the effect of etiology in the present cohort. Future studies should therefore include comparable tooth types across etiological groups, standardized assessment of trauma characteristics and root developmental stage, and appropriate statistical approaches to account for potential confounding factors.

Although a standardized paralleling technique and the Rinn XCP alignment system were used and the images were calibrated using a reference scale, minor differences in projection geometry and magnification between preoperative and follow-up radiographs cannot be completely excluded and may have introduced measurement error. In addition, factors such as radiographic exposure parameters, sensor positioning, and focus-to-receptor distance may have varied between clinical examinations and could not be completely controlled in this retrospective study. Furthermore, Microsoft PowerPoint was used as the measurement platform rather than dedicated radiographic image-analysis software, which may have introduced additional measurement variability and should be considered when interpreting the quantitative radiographic outcomes.

An additional methodological limitation concerns the two-dimensional nature of the radiographic assessment. Periapical radiographs provide a projection of three-dimensional anatomical structures and therefore cannot fully characterize the circumferential and longitudinal changes that occur during root maturation. In the present study, dentinal wall thickness was measured at a predefined level corresponding to two-thirds of the preoperative root canal length. Although this approach allowed a standardized and reproducible comparison between baseline and 12-month radiographs and has been used in previous studies evaluating regenerative endodontic outcomes, a single linear measurement at one root level may not fully represent the overall changes in dentinal wall thickness throughout the root. Three-dimensional imaging techniques, such as cone-beam computed tomography (CBCT), may provide a more comprehensive assessment of root maturation by allowing volumetric evaluation of root, dentine, and pulp space changes. However, CBCT examinations were not available for all patients in this retrospective cohort and could not have been obtained solely for research purposes without an independent clinical indication, particularly considering the additional radiation exposure associated with three-dimensional imaging in pediatric patients [25]. Future prospective studies should therefore consider three-dimensional assessment when clinically justified. Alternatively, when only periapical radiographs are available, measurements at multiple standardized levels along the root could provide a more comprehensive characterization of dentinal wall changes and potentially reduce the influence of localized variations in root development.

From a clinical perspective, the findings of this study describe favorable clinical and radiographic outcomes following regenerative endodontic treatment in immature permanent teeth with pulp necrosis. However, because all teeth received the same treatment and no control or comparison group was included, the present study cannot establish treatment effectiveness or superiority compared with alternative treatment approaches. The retrospective design, small sample size, short follow-up period, absence of a control group, potential radiographic measurement variability, and substantial differences in tooth type and developmental stage between the etiological groups further limit the generalizability and causal interpretation of the findings. Therefore, the observed differences between the groups should be interpreted as associations rather than as evidence of an etiological effect. In particular, the greater dentinal wall thickening observed in the carious group may reflect differences in tooth type, developmental stage, trauma characteristics, or other factors that could not be fully assessed, rather than an effect of pulp necrosis etiology alone. Further prospective studies with larger samples, comparable tooth types across etiological groups, standardized assessment of trauma severity and root developmental stage, appropriate control or comparison groups, longer follow-up periods, and standardized radiographic or three-dimensional assessment are needed to determine whether pulp necrosis etiology independently influences the long-term outcomes of regenerative endodontic treatment.

## 5. Conclusions

Considering the limitations of the present study, the following conclusions can be drawn:

- In this retrospective cohort, favorable clinical and radiographic outcomes were observed following regenerative endodontic treatment in immature permanent teeth with pulp necrosis.
- Both groups showed continued root development during the 12-month follow-up period. No statistically significant difference was observed in the percentage change in root length between the groups, whereas the carious group showed a significantly greater increase in dentinal wall thickness. However, because the etiological groups differed substantially in tooth type and stage of root development, and because other factors such as trauma characteristics could not be fully assessed, these differences cannot be attributed solely to the etiology of pulp necrosis.
- All treated teeth showed radiographic resolution of periapical pathology at the 12-month follow-up, although the pattern and extent of root development varied according to the individual case.
- These findings should be interpreted cautiously because of the retrospective design, small sample size, absence of a control or comparison group, limited follow-up period, and potential variability in radiographic measurements. Larger prospective studies with standardized radiographic protocols, longer follow-up, and appropriate comparison groups are needed to determine whether the etiology of pulp necrosis influences the long-term outcomes of regenerative endodontic treatment.

## Figures and Tables

**Table 1:** Eligibility criteria for the selected study sample.

Inclusion Criteria	Exclusion Criteria
Patients without systemic diseases	Teeth with vertical fractures
Preoperative radiographs showing an immature apex accompanied by apical periodontitis	Teeth with periodontal conditions
Radiographic assessment at 12 months after treatment.	Non-restorable teeth

**Table 2:** Summary of root length, root wall thickness and outcome with after endodontic regenerative with traumatic etiology.

Case	Tooth	Preoperative Cvek’s Stage of Root Development	Root Length % Change (12 Months)	Dentin Thickness % Change (12 Months)	Type of Repair	Outcome
1	21	3	22.2%	20%	Periodontal	Healed
2	11	2	1.19%	20%	Periodontal	Healed
3	21	3	11.67%	11.11%	Periodontal	Healed
4	21	3	2%	33.33%	Periodontal	Healed
5	21	3	2.67%	33.33%	Periodontal	Healed
6	11	3	2%	33.33%	Periodontal	Healed
7	21	2	4%	0%	Periodontal	Healed
8	11	2	33.33%	22.73%	Periodontal	Healed
9	21	3	3.1%	5%	Periodontal	Healed
10	11	2	17.02%	18.8%	Periodontal	Healed

Note: Cases were classified as showing predominantly pulpal or periodontal repair based on the observed pattern of hard-tissue formation and root development.

**Table 3:** Summary of root length, root wall thickness and outcome with after endodontic regenerative with carious etiology.

Case	Tooth	Preoperative Cvek’s Stage of Root Development	Root Length % Change (12 Months)	Dentin Thickness % Change (12 months)	Type of Repair	Outcome
1	16	3	10%	71.43%	Pulp	Healed
2	36	4	10%	14.29%	Pulp	Healed
3	46	3	31.25%	12.5%	Pulp	Healed
4	46	3	3.23%	36.36%	Pulp	Healed
5	46	4	10.24%	81.82%	Pulp	Healed
6	36	4	9.41%	77.8%	Pulp	Healed
7	36	3	31.25%	50%	Pulp	Healed
8	26	4	14.29%	72.73%	Pulp	Healed
9	36	3	25.08%	12.5%	Pulp	Healed
10	46	4	15%	60%	Pulp	Healed

Note: Cases were classified as showing predominantly pulpal or periodontal repair based on the observed pattern of hard-tissue formation and root development.

## Data Availability

The data analyzed in this study were obtained retrospectively from existing clinical and radiographic records. The data are not publicly available because they consist of patient-derived clinical and radiographic information and are subject to confidentiality and data protection requirements.
